# Signal processing for molecular and cellular biological physics: an emerging field

**DOI:** 10.1098/rsta.2011.0546

**Published:** 2013-02-13

**Authors:** Max A. Little, Nick S. Jones

**Affiliations:** 1 MIT Media Lab, Room E15–390, 20 Ames Street, Cambridge, MA 01239, USA; 2 Department of Mathematics, Imperial College London, South Kensington Campus, London SW7 2AZ, UK

**Keywords:** biophysics, molecules, cells, digital signal processing

## Abstract

Recent advances in our ability to watch the molecular and cellular processes of life in action—such as atomic force microscopy, optical tweezers and Forster fluorescence resonance energy transfer—raise challenges for digital signal processing (DSP) of the resulting experimental data. This article explores the unique properties of such biophysical time series that set them apart from other signals, such as the prevalence of abrupt jumps and steps, multi-modal distributions and autocorrelated noise. It exposes the problems with classical linear DSP algorithms applied to this kind of data, and describes new nonlinear and non-Gaussian algorithms that are able to extract information that is of direct relevance to biological physicists. It is argued that these new methods applied in this context typify the nascent field of biophysical DSP. Practical experimental examples are supplied.

## 1. Introduction

Molecular and cellular biological physics is interested in the physical mechanisms that make up life at the smallest spatial scales [1]. A large part studies the mechanisms that lead to changes in the configuration of a molecule or sets of interacting molecules, which have biochemical consequences when these molecules are present in large numbers. Whereas a biochemist might describe F1-ATPase as an enzyme that accelerates the production of the substance adenosine triphosphate (ATP), the biological physicist might say that it is a molecular rotary motor, driven by a proton gradient, and each single proton binding event causes a 120^°^ rotation of the motor, which in turn causes an ATP molecule to be released.

In recent years, biological physicists have developed numerous experimental tools that provide unprecedented insight into the real-time, molecular basis of the chemical processes of life. These measurement techniques record the dynamic changes in configurations of molecules or sets of interacting molecules, such as protein assemblies. In some cases, the experiments are conducted on living cells, in others, on isolated molecules or molecular assemblies. Very often the measurement is a time series or digital signal that can be processed using signal processing algorithms. These algorithms extract, from the time series, quantities of interest to the experiment.

Some of the questions experimentalists want to ask can be addressed using classical, linear digital signal processing (DSP) tools applied to the resulting measurements. Yet, important questions cannot be answered using classical tools. Part of the reason for this is that time series from these biophysical experiments have peculiar properties that make them quite unlike signals from other scientific domains. For example, abrupt transitions are pervasive because the dynamics of molecular motion often occurs in a sequence of small steps, as this makes the optimum use of the available free energy stored in molecular bonds [1]. So, this requires the use of non-classical nonlinear and/or non-Gaussian signal processing algorithms. These algorithms are often interesting in their own right as they provide examples where advances in DSP have novel practical applications in science.

Biophysical signal processing algorithms have been developed by experimentalists to solve problems specific to their own questions of interest. Because of this, the focus has not been on the theoretical issues that arise in processing generic biophysical time series across disciplines, and the relevant examples are scattered across disparate literature, including physics, chemistry, neuroscience, nanotechnology as well as biological physics. We now provide a few examples with an orientation towards the detection of steps (in §2 we will discuss why this is a particular concern, and in §4 we will summarize some of the methods that are used). To enhance the detection of steps in the force generated by kinesin molecular motors, Higuchi *et al.* [2] applied the nonlinear, running median filter to force–time traces measured using atomic force microscopy. Sowa *et al.* [3] used an iterative, nonlinear step-fitting technique (originally developed to characterize the growth of microtubules [4]) to provide direct observational evidence for discrete, step-like motion in time–angle traces of the bacterial flagellar motor (BFM). Influenced by the study of neuronal firing currents, a nonlinear adaptation of the running mean filter was derived [5], explicitly to address the problem of smoothing in the presence of abrupt jumps. The resulting algorithm has found applications in the study of DNA polymerase conformational changes [6], in the analysis of fluorophore emission time series in cell membrane fusion processes [7] and in examining the intermediate steps making up ribosome translation [8]. A largely complementary signal processing approach is the use of hidden Markov modelling (HMM) [9], among many other experimental applications, to studies of ion-channel currents [10], the conformational changes of Holliday junctions and monomer DNA binding and unbinding measured using single-molecule fluorescence energy transfer [11], and the dynamics of molecular motors [12]. Finally, the running *t*-test has been applied to problems such as nanopore DNA sequencing [13].

However, with a few exceptions [14–17], no widely inclusive attempts have been made to discuss generic characteristics of biophysical signals, and the common mathematical principles behind the design of the disparate algorithms used to investigate their step-like character have received little attention. On the empirical side, these algorithms have rarely been tested head-to-head. The main purpose of this article is to present some conceptual groundwork for the study of signals generated by discrete (molecular) systems.

An outline of the article is as follows. We describe the particular properties of some molecular and cellular time series that set them apart from other signals in §2. This is followed by a description of some of the more popular experimental assays in §3. Then, in §4, we review a select range of time-series analysis methods that are used in biological physics experiments. Finally, §5 explores some examples of specific physical experiments where DSP tools are used to answer questions of biological importance.

## 2. The distinctive character of molecular and cellular processes

At the molecular scale, motion is dominated by thermal fluctuations and diffusion: life has evolved to be both robust against and to exploit this disorder. Of central importance to living processes are proteins: long chains of molecules, mostly tightly folded into particular configurations, which interact with other proteins in a complex web of biochemical reactions. Many proteins, once constructed, partly lock together into self-contained assemblies within the cell, which together go through sequences of configurations to achieve a certain end product. Other proteins diffuse freely or are transported within the cellular environment, binding with specific atoms, molecules and assemblies, as and when they encounter them. Whether in motion or locked together, the changes in configuration that proteins undergo are always subject to thermal fluctuations. This means that most data recorded from molecular-scale biophysical experiments are, usually, noisy, and one major challenge of biophysical signal processing in this context is how to remove this noise (whose physical nature is sometimes well characterized) leaving only the relevant biophysical dynamics.

### (a) Langevin dynamics

**Figure 1.**
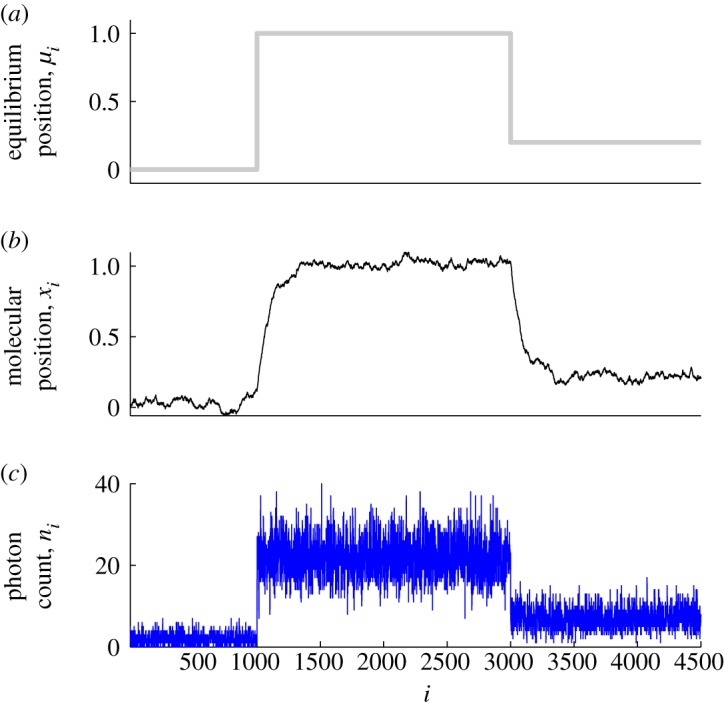
Simulated traces illustrating the unique characteristics of biophysical time series. (*a*) Step-like transitions; the molecular system switches between equilibrium states on a time scale faster than the experimental sampling interval. (*b*) Langevin dynamics and autocorrelated noise; the effect of thermal Gaussian noise and experimental apparatus creates ‘rounding’ of the state switching, and introduces Gaussian stochastic fluctuations around the measured state. (*c*) Poisson photon count noise; experiments often use light to make recordings and so the random emission of photons causes stochastic fluctuations in the digital signal, the spread of the fluctuations increasing with the intensity of the detected light. In each panel, the horizontal axis is the sample index. (Online version in colour.)

An approximate model of the behaviour of a molecular system subject to thermal noise is a linear second-order stochastic differential equation with inertial, frictional (or drag) and potential terms [15]. The stochastic input is Brownian motion. The second-order, inertial term is usually considered to be small because the ratio of the mass to the coefficient of friction (or drag) is small. This leaves a first-order differential system that describes the motion owing to potential, friction and thermal collision forces:
2.1
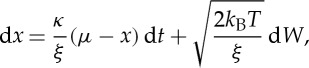
where *x* is the position of the molecular system, *κ* the strength of the potential, *ξ* the coefficient of friction, *μ* the equilibrium position of the system, *k*_B_ Boltzmann's constant, *T* the temperature of the surrounding medium and *W* a Wiener process. This equation has an Ornstein–Uhlenbeck process as solution, and is useful in a range of experimental settings. In practice, the model is solved using a numerical method: in §4, we will describe a particular experimental setting in which a discretized Langevin model is used to represent an experiment studying a BFM, and this model is sufficiently simple that it can form the basis of a signal processing method to extract the arrangement and sequence of rotational changes in the motor. Langevin dynamics are illustrated in figure 1*b*.

### (b) Poisson and Gaussian noise

Light is a fundamental tool in the experimental study of molecular systems and can be generated by molecular probes such as fluorophores in response to laser illumination (see §3). The duration between emissions of individual photons is approximately exponentially distributed, so that the experimental noise (*shot noise*) is a Poisson point process. However, thermal noise appears to be well modelled as Gaussian [18].

Removal of Poisson noise formally requires signal processing methods that are adapted to the specific distribution of this noise: one peculiarity of Poisson noise is that the variance of the noise increases with the photon count, which means that in certain experimental settings the variance depends on the molecular configuration. By contrast, for Gaussian thermal noise, the variance is largely independent of the molecular configuration. In bright illumination or emission settings, the large numbers of photons involved cause the shot noise distribution to approach a Gaussian. Then, the photon noise distribution is also normally distributed.

In certain situations, for example, in fluorescence resonance energy transfer (FRET; see §3*b*), the observed photon count, *n*, is Poisson distributed with mean parameter that is a decreasing function *f*(*r*) of the separation between the donor and acceptor fluorophores *r* which are used to measure the configuration of the molecular system:
2.2
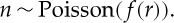
Assuming that *n* remains large enough and the configuration of the system changes smoothly, *r* can be adequately recovered from the observed photon count signal *n* using classical linear signal processing tools. Figure 1*c* depicts the typical effect of Poisson photon count noise.

### (c) Step dynamics

In the experimental assays described below (see §3), signals from molecule-scale experiments are often only available at sampling rates down to the time scale of milliseconds. But, molecular systems of interest, particularly small ones, can undergo configuration changes orders of magnitude faster than this rate [19]. Thus, these changes can appear to be instantaneous when looking at the recorded digital signal (figure 1*a*). This non-smoothness poses special challenges for classical, linear signal processing techniques, such as smoothing filters, which aim to remove experimental noise from recorded data. It is therefore necessary to use nonlinear and non-Gaussian analysis tools instead (§4).

The molecular system under study may go through a sequence of different configurations. These sequences of configurations may have no temporal dependence, but sometimes there are good biophysical reasons to consider this sequence to be generated from a Markov chain. Then the signal processing goal is to determine the transition density and initial densities of the chain from the data, and under certain assumptions, tools such as HMM are useful. We will see in §4 how HMM and other nonlinear or non-Gaussian signal processing tools for analysing step dynamics are related.

### (d) Autocorrelated noise

Thermal noise, when recorded in digital signals, appears independent. This is convenient because, from a technical point of view, it simplifies the process of noise removal. Nonetheless, experimental apparatuses for studying molecular systems are very complex, and it is quite possible to introduce time dependencies into the signal that do not originate in the molecular system. For example, laser-illuminated gold beads that can be fixed to a protein assembly in order to record motion have large mass and therefore introduce spurious momentum into the experiment. The bead is driven by independent thermal noise as well as the dynamics of the protein assembly, so the recorded signal shows significant autocorrelation whose decay rate is partly a function of the bead mass (see figure 1*b* for an illustration of this phenomenon). Much more care in the signal processing has to be applied in order to remove this kind of noise. We will see in §5 an example where combining a discrete stochastic differential equation model with a nonlinear step detection algorithm achieves good results on autocorrelated noise.

## 3. Experimental assays and data pre-processing

### (a) Single-molecule force spectroscopy

The atomic force microscope is a high-precision instrument for quantifying interaction forces at the atomic and molecular scale [20]. A cantilevered tip of nanometre proportions is brought into close proximity to a sample and the tip is deflected by chemical bonding, capillary, electrostatic or other forces. The tip deflection, which is of the order of nanometres, is amplified through the cantilever, and this amplified motion is measured in real time using, for example, a laser, the changing path of which is recorded optically. The typical scale of forces involved is of the order of piconewtons (10^−12^ N) and upwards. A molecular sample is attached to a mount which can be moved using piezoelectric motors.

Atomic force microscopy has been adapted for use in single-molecule biophysical experiments. In particular, it has been used to measure the time dynamics of forces involved in receptor–ligand recognition and dissociation [21]. A molecule is attached to a surface using, for example, thiols, which have much larger covalent bonding force than recognition binding. The experimental output is a direct digital measurement of the deflection of the laser spot, which can be related back to the force over time during the recognition or unbinding events.

### (b) Forster resonance energy transfer

FRET-based microscopy is, primarily, a technique for measuring distances at the nanometre scale between atoms and molecules. It is based on fluorophores: light-emitting and light-sensing molecules such as the naturally occurring green fluorescent protein and derivatives, or quantum dots which are entirely synthetic. The basic experimental approach is to attach (‘tag’) fluorophores to individual molecules or molecular assemblies, and then monitor changes in light emitted by these fluorophores as the tagged molecules interact or change conformation over time. Tagging can be achieved using a variety of methods, including genetic engineering.

The FRET process involves one or more pairs of donor and acceptor fluorophores which exchange energy optically. In FRET, the donor must be excited by an external illumination source. The efficiency of this energy exchange can be imaged with an optical microscopy set-up, usually captured using an electron-multiplying charge-coupled device (EMCCD) running at high imaging frame rates (1 kHz or more). The resulting sequence of EMCCD FRET images are digitally analysed (see §4*e*) to produce a pair of time series that together determine the FRET efficiency signal. There is a direct, inverse power-law relationship between FRET efficiency and the donor–acceptor separation distance, and this can be used to infer changes in distance between the donor- and acceptor-tagged molecules under study.

### (c) Laser-illuminated beads

Cellular or molecular systems of interest to biological physicists are usually too small to image directly. An alternative solution to fluorescence or atomic force microscopy is to attach an object to the system under study that is sufficiently large to be imaged directly. Popular objects are microspheres such as fluorescent polystyrene or gold, of size 10–1000 nm in diameter. These can be attached to the system using linkers, typically made from biotin or streptavidin. The beads naturally place some load onto the system under study that must be considered in the analysis (see §5). The bead is then mechanically connected to the system under study, and the moving bead can then be directly imaged. Typically, the bead is laser illuminated in order to provide good contrast in an EMCCD-captured microscopy set-up. Examples of such illuminated bead assays include monitoring the rotation of the BFM [15,22] and the rotation of F1-ATPase enzyme [15].

### (d) Charge-coupled device image pre-processing

As described above, light plays a critical role in many molecular or cellular experiments, and one of the most common measurement tools of this light is the high-speed EMCCD video camera. This captures a sequence of images obtained using a microscope, often at high frame rates of up to 1 kHz. The goal is to process these frames to produce a time series that contains the information of relevance to the experiment. For example, in FRET experiments, images of the fluorophores are captured, and the intensity of the fluorophores is used to infer the FRET efficiency. At that physical scale, it is reasonable to consider them as point source emitters [23]. Because the imaging system is linear and time-invariant (see §4), the captured image represents the point source convolved with the point spread function of the optics. The point spread function is usually modelled as a two-dimensional, isotropic Gaussian [24]. Extracting the time change in intensity of each fluorophore involves fitting this Gaussian to each frame of the video, which is usually corrupted by uniform background illumination noise. For the isotropic point spread function, there are, at the minimum, three parameters to optimize the horizontal and vertical location, and the variance. The location parameters can have better precision than the image resolution, which allows super-resolution localization of the fluorophore. A recent study has identified the maximum-likelihood estimate for these parameters leading to the best performance under controlled conditions [23].

## 4. Signal processing

### (a) Noise removal and step detection: the inadequacy of linear filtering

One of the ‘canonical’ problems in signal processing is filtering: the removal of some component of the signal while leaving the other components unchanged. Configuration changes in molecular and cellular systems are obscured by thermal and other sources of noise. The classical, linear signal processing solution to this problem is *smoothing* or *filtering*: by obtaining (weighted) averages over a temporal window around each sample in the signal, a statistical estimate of the configuration at each point in time can be obtained. However, severe limitations to this strategy arise when the signal can change abruptly, rather than smoothly: in fact, this is not a problem for which classical linear DSP is suited.

To illustrate why classical linear filtering is problematic in this situation, consider the archetypal, step-like signal: the *square wave—*periodic with instantaneous transitions between two different amplitudes—which is obscured by serially uncorrelated (white) noise. A fundamental fact about linear, time-invariant (LTI) systems is the existence of a unique spectral description, so it is instructive to describe the situation in the Fourier domain. The only Fourier coefficients of the square wave that are non-zero are odd, integer multiples of the frequency of the wave, and are proportional to 1/*n*, where *n* is the index of the Fourier component. Thus, the Fourier series has an infinite number of non-zero terms (infinite bandwidth), and truncating the series introduces spurious oscillations (Gibbs phenomena) near the edges of the square wave, with the amplitude of these oscillations increasing as the truncation becomes more drastic. At the same time, serially uncorrelated (white) noise has constant spectral density, so the bandwidth of the noise is also infinite.

The LTI smoothing filter averages over a certain duration of time (low frequency), in order to integrate over statistical fluctuations due to the noise occurring on a smaller time scale (high frequency). In the Fourier domain, therefore, the filter recovers low-frequency signal by removing high-frequency noise, but this only works in principle if the signal does not have any non-zero, high-frequency Fourier coefficients. Therefore, an LTI filter can never completely separate abruptly changing signals from uncorrelated noise, because both have infinite bandwidth. This is unfortunate because if we consider the common experimental case of molecular dynamics obscured by large-count photon noise, then the simple, running mean filter achieves the minimum mean-squared error of all estimators of the underlying configuration, if it is static (because the large-count photon noise is nearly Gaussian, see §2*b*, and the sample mean is the minimum variance unbiased estimator of the underlying position at the mean of the Gaussian).

Nonetheless, the only way to increase the accuracy of the filter is to extend the time duration, that is, to integrate over a larger temporal window. This increases the truncation of the Fourier series, which exacerbates the unwanted Gibbs phenomena. Another side-effect of this window size increase is to ‘smear out’ the precise time localization of any configuration changes in the wanted signal. But, since a square wave is defined completely by the time localization of its transitions, and the value of the amplitudes, an LTI filter must inevitably trade the accuracy in the estimate of the amplitude of the signal against the accuracy of the time localization of the abrupt changes. There exist non-LTI filters that can achieve different, and usually more useful, trade-offs in this respect, which we discuss next.

### (b) Median filtering and other nonlinear running filters

The nonlinear, running median filter has found extensive use in biological physics experiments [2,17,22,25,26]. This filter uses the median in a temporal window surrounding each time point as an estimate of the wanted signal. The running median has certain desirable properties: it is straightforward to show that any abrupt changes in a noise-free signal pass through the filter unaltered, whereas the LTI filter must smear out these transitions, even for signals without noise [27]. Nonetheless, for a given temporal window size, the median filter is not as efficient at removing noise from constant signals as the mean filter. If the noise is Gaussian, the median filter will be outperformed by a mean filter of the same window size, but it will achieve far more precision in the estimate of the time location of the transitions. Furthermore, even a single experimental outlier can critically distort the output of the running mean filter, whereas the median filter is robust to outlier corruption in up to half of all the data in each temporal window [28].

Other running nonlinear filters employ a variety of schemes to improve this ‘smearing–smoothing’ trade-off inherent to running mean and median filters. One example is data-dependent weighting [16], for example, a weighted running mean filter, where the weights depend upon the heights of any transitions in the recorded signal. This is useful because, to a large extent, it avoids filtering over large abrupt changes [5,16]. This works well if the size of the abrupt transitions is large by comparison to the spread of the noise, but this situation does not often occur under realistic experimental conditions.

If an approximate value of the measured signal for each stable configuration of the biophysical system under study is known, this information can be incorporated into an efficient Bayesian running median filter [29]. The result is a filter that has far better performance than the standard running mean or median filter, but it is rare to have this kind of prior information in practice.

Another approach that finds common use in biophysical experiments are running window statistical hypothesis tests, the classical example of this being the running *t*-test [13,14]. This operates under the assumption that if any temporal window contains a transition, one half of the data will have a different mean to the other half, and this difference in mean can be detected using the two-sample *t*-test. The major limitation to this strategy is that it assumes the existence of at most one transition within each window. The statistical power of the test is improved by increasing the temporal window size, but, as well as decreasing temporal resolution, this risks the situation where the window contains more than one transition that renders the assumptions of the test invalid. Therefore, there is an unavoidable ‘power–validity’ trade-off.

In the next section, we will describe approaches to the problem of noise removal that altogether sidestep these trade-offs, that originate primarily in the use of temporal windowing.

### (c) Piecewise constant models: spline and level set recovery

The prevalence of the use of running filters for noise removal from time traces in biological physics experiments may have its origins in filtering as the most intuitive and obvious method. These have the virtue of being extremely simple, but they lack the sophistication required to process many biophysical signals effectively.

A good model for the abrupt switching between stable configurations seen in many biophysical systems is a constant spline [17]. A spline is a curve made up of continuous (and usually also smooth) segments joined together at specific locations, called knots. In a biophysical signal with abrupt transitions, the knots are located at the transitions and the continuous segments are constant, representing each stable configuration (figure 1*a*). An entirely equivalent representation is in terms of level sets [17]. In this model, each stable configuration is associated with a unique constant value and the time intervals (level set) where the biophysical signal assumes that value. Both of these models are piecewise constant, and lead us away from the view that the problem of noise removal from typical biophysical experimental time series is a smoothing problem: it is more accurately described as an exercise in recovering piecewise constant signals obscured by noise [17].

In recovering a level set description, the time location of the transitions can usually be determined once the values of the stable levels have been recovered. Algorithms more traditionally studied in the machine vision and statistical machine learning literature are well suited to this task, in particular, clustering using *K*-means, mean shift or Gaussian mixture modelling (GMM). By contrast, methods which find the time location of transitions first from which values of constant levels can be inferred include stepwise jump placement and total variation regularization.

The majority of these piecewise constant noise removal algorithms can be formalized under a generalized mathematical framework, which involves the minimization of a functional equation [17]:
4.1
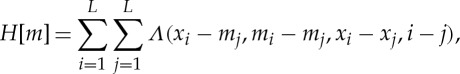
where *x* is the observed signal of length *L* input to the algorithm, and *m* is the piecewise constant output of the algorithm, also of length *L*. The function *Λ* determines the specific kind of noise removal technique. The functional equation (4.1) can be minimized by an appropriate algorithm that varies *m*. Which type of algorithm depends on the choice of *Λ*. For example, if the resulting functional is *convex* in the first two parameters (those that involve *m*), then standard methods such as linear or quadratic programming can be used [30]. Alternative iterative methods such as jump placement or adaptive finite differences can be used in cases where equation (4.1) is non-convex [16].

An important special case of equation (4.1) is
4.2
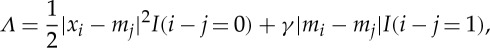
where *I*(*S*)=0 if the logical condition *S* is false and *I*(*S*)=1 if *S* is true. This defines total variation regularization, which is a popular method for digital image processing [17,31]. The regularization constant *γ* is related to the product of the time difference (in samples) between transitions and the size of the transitions. More precisely, for a constant region of the signal lasting *w* samples between transitions of height *h*, if *γ*>(*w* *h*)/2, this constant region will be smoothed away by merging with neighbouring constant regions [17]. Therefore, as with the window size in running filters, the larger the parameter *γ*, the larger the combined time/amplitude scale of features to be removed. Unlike running filters, however, the smoothing occurs by shrinking the size of transitions until the constant regions they separate are merged together. This means that the output of the algorithm, *m*, is always piecewise constant, which is a desirable property for biophysical time series.

The output is easily modelled as a constant spline, whose knots are removed in sequence as *γ* increases, the corresponding constant intervals adjacent to each knot being joined together into a new interval whose value is the average of the two intervals. Finally, by the same logic, if the noise to be removed is Gaussian with standard deviation *σ*, setting *γ*>2*σ* smoothes away approximately 95 per cent of the noise on average. Of course, any wanted feature in the signal whose combined time/amplitude scale is less than *γ* will also be smoothed away, and so there is a trade-off between noise removal and retention of small features on the same scale as the noise.

The total variation regularization functional equation (4.1) obtained by applying equation (4.2) is in quadratic form, and can be efficiently minimized using quadratic programming [30]; alternative algorithms include piecewise constant spline LASSO regression and coordinate descent [16].

Another very useful special case of equation (4.1) is
4.3
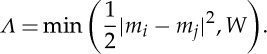
This defines *mean shift*, a ubiquitous clustering technique [17], and it can be understood as a method for performing level set recovery from a noisy signal. Since this function is independent of *x* its minimizer, *m*, is only non-trivial if we put a constraint on the method of minimizing it. In this case, *m* is initialized by setting it to *x* and various iterative procedures are used to lower the value of equation (4.1). The classic mean shift algorithm takes the original signal as its initial condition and then iteratively replaces each sample in the data with a weighted mean of all the other samples until no improvement is shown: the weighting depends upon the difference in value between each sample. One can show that this procedure is a minimizer for equation (4.1) when *Λ* is as equation (4.3) [16]. The mean shift algorithm cannot increase the functional equation (4.1), and so it eventually converges, and typically, the output signal *m* will be piecewise constant. Convergence is usually fast, occurring after only a few iterations [32].

The parameter *W* gives some measure of control over scale of separation of level sets. If *W* is large, the constant value associated with each level set will be well separated from the others. The trade-off is that the number of different constant levels tends to be inversely proportional to the separation between them. If the separation between levels is not homogeneous, then closely spaced levels can become merged erroneously, or single levels that have large noise can become erroneously split into multiple levels.

A wide range of piecewise constant recovery methods with differing properties can be obtained using slight variations in the form of *Λ*. If the noise deviates from Gaussianity (as an example, consider the case of low-count photon noise) it may be better to use robust total variation regularization [16]:
4.4
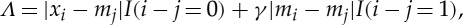
where the first term, the square error, has been replaced by the absolute error. The resulting functional equation (4.1) is convex and can be minimized using standard linear programming algorithms.

### (d) Markov chain analysis

We have seen above that the problem of noise removal from typical biophysical experimental time-series data is usually best understood as a problem of recovering of a sequence of constant levels with instantaneous transitions, hidden by experimental noise. Each constant level represents a distinct, stable conformational state of the molecular system. Many molecular and cellular systems undergo sequences that have no temporal dependence: the next conformational state may depend upon which state it is in currently. This can be modelled as a Markov chain. Therefore, one important experimental goal is to find the parameters of the chain (the transition probabilities) when the state of the biophysical system is obscured by experimental noise. This is a classical problem in signal processing known as hidden Markov models (HMM), and it has found extensive use in interpreting biophysical experiments [11,12,33–37].

There are many variations on the basic HMM algorithm. However, most exploit one of the key concepts making HMM popular in practice: the existence of a simple algorithm to estimate the probability of any given sequence of states, and/or the sequence of most probable states. This leads to a version of the expectation–maximization (EM) algorithm that iteratively estimates the HMM parameters by alternately calculating the state probabilities followed by the noise distribution parameters [38]. Because the likelihood surface for the HMM is non-convex, EM finds a local optimum which is not necessarily the global one.

In biophysical contexts, it is common to assume that the observation noise is Gaussian, which makes the HMM distribution parameter estimates straightforward. We can give the following formal description of the Gaussian HMM. The molecular state *y*_*i*_ at time sample *i* takes one of the numbers 1,2,…,*K*, where *K* is the number of states: each state corresponds to a different configuration of the system. The transition probabilities are contained in the *K*×*K* matrix P. Finally, the observed signal *x*_*i*_ is drawn from one of *K* Gaussians with means *μ*_1,2…*K*_ and standard deviations *σ*_1,2…*K*_, so that
4.5
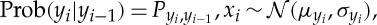
where 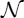 refers to the Gaussian distribution, and ‘∼’ means ‘distributed as’. HMMs with discrete states require the number of states to be chosen in advance. This is not always desirable and so the number of states usually needs to be regularized. A brute-force approach to regularized HMM fitting involves repeatedly increasing the number of states and re-estimating the likelihood of the fit. A simple approach to regularizing with respect to the number of states combines the negative log likelihood (NLL) of the HMM given estimated values of the parameters with the Akaike information criterion [39]:
4.6
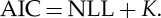
The number of states *K* leading to a minimum in equation (4.6) is taken as the correct number of states.

HMMs are special kinds of Bayes networks [38], which include classical signal processing methods, such as the Kalman filter, but also clustering methods, such as the Gaussian mixure model (GMM). In fact, the Gaussian HMM popular in biophysical contexts can also be understood as a time-dependent version of the GMM. This also connects with the clustering described above, in that *K*-means clustering can be seen as a special case of the GMM where the most probable assignment of time samples to clusters is used rather than the probability of each sample belonging to a cluster as in the GMM [16].

The EM algorithm is very general. For example, the noise in many biophysical experiments is often highly autocorrelated (see §2*d*). The maximization step in EM can often be performed using closed-form calculations. In particular, if it is assumed that the observations are generated by an autoregressive linear system, then the parameters of the linear system can be estimated in closed form using matrix algebra. This gives a simple approach to estimating states hidden behind autocorrelated experimental noise [33].

EM is not the only way to perform parametric inference in HMMs, but it is the most common tool. Other approaches involve direct minimization of the negative log likelihood using numerical gradient descent [34]. It should, however, be mentioned that, flexible though the HMM framework is, it has a lot of adjustable parameters and the likelihood surface for parameter optimization can be challengingly non-convex. What this implies is that any one set of parameter values, obtained at convergence, cannot be used with confidence, because it is computationally challenging to know whether these lead to the global optimum of the likelihood surface. One partial solution is to run an iterative parameter inference algorithm to convergence from a randomized set of initial parameter values. Then, the converged set of parameter values that lead to the largest likelihood can be used.

A disadvantage with the direct use of HMMs is the strong assumption that the signal is generated by a fixed number of recurring states with means *μ*_1,2…*K*_. It is entirely reasonable to have experimental systems which appear to show a continuum of states (e.g. if the levels of the HMM might themselves undergo a random drift because of an experimentally uncontrolled systematic variation).

### (e) Non-parametric estimates for periodic distributions: histograms, kernel densities, characteristic functions and shrinkage

A common problem in most scientific domains is estimating the distribution of sampled data. In biophysical experiments, it is often important to know the distribution of states, because this can tell us how many discrete states there are, and their relative spatial separation. Estimating distributions from data is a central topic in statistics and has been studied extensively. Here, we are concerned with the situation where very little about the mathematical form of the distribution can be assumed in advance, which leads to the area of statistics known as non-parametric distribution estimation.

One of the simplest approaches still finding a lot of use in biophysics is the histogram. The histogram involves choosing a bin spacing and the left-most bin edge. Then, the number of experimental data points that fall within each bin is counted. Normalizing this count in each bin by the number of samples builds a valid distribution for the data [40]. There are many difficulties that arise with this approach, however. In particular, the results are highly sensitive to the number of bins chosen, with small bin spacing often leading to the count in each bin becoming sensitive to the sampling variability of the data. At the other extreme, picking a small number of bins leads to large bin spacings that tend to smooth off real variability in the shape of the distribution. Standard approaches to selecting bin width are detailed in [41].

This trade-off between high sensitivity to sampling variability and tendency to smooth away genuine fluctuations in the form of the distribution is intrinsic to density estimation with histograms [40]. One alternative approach that has certain advantages over the histogram is the kernel density estimate (KDE). The KDE is, fundamentally, a ‘smoothing’ approach to distribution estimation. Even though a density function can be defined (consistently) by a finite number of samples of the associated random variable, this function is, by construction, non-smooth, consisting of an equally weighted series of (Dirac) delta functions placed at each sample *x*_*i*_:
4.7
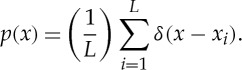
The KDE convolves a smooth kernel function *κ* with the delta density function equation (4.8) to produce a smooth density estimate
4.8
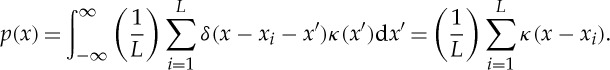
The convolution can be efficiently carried out in the Fourier domain by discretizing the domain of the random variable and using the fast Fourier transform [40].

The KDE circumvents the problems of non-smoothness inherent to histograms. A typical choice of kernel is the Gaussian density function, which has a single, standard deviation parameter. If that parameter is too large, then the KDE risks smoothing away real fluctuations in the density function: if it is too small, sampling variability will cause the KDE to fluctuate spuriously. Of course, there is flexibility in choosing the form of the kernel unlike histograms, but with any smooth, symmetric density kernel, the choice of kernel width presents a similar trade-off as in the choice of histogram spacing: one must then turn to established methods for selecting bin-size kernel width [40].

Many molecular or cellular systems have the property that they consist of a series of interlocking proteins or other assemblies that have a *periodic structure*. For example, the BFM consists of several rings of proteins in a periodic arrangement that function as a ‘stator’, within which another assembly (the ‘rotor’), also made from periodic protein rings, rotates. Because of this structural periodicity, the arrangement of stable configurations of the motor assembly is also periodic. This means that any distribution estimate of the rotation of the motor needs to be able to pick out this repetition in the spacing of the peaks (*modes*) of the distribution. There may be more than 20 modes, they may not be equally spaced and they will have different heights (corresponding to the different amounts of time spent in each conformational state) [3,15].

This is a multi-modal distribution and the large number of modes causes the GMM method to have a challenging non-convex likelihood function, which makes GMM parameter estimation unreliable. However, since we know that the distribution is periodic, we can use the probabilistic equivalent of the Fourier transform, the empirical characteristic function (ECF), to estimate the distribution instead:
4.9
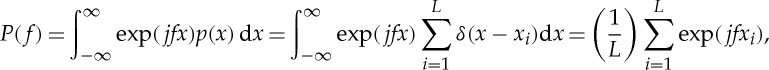
where 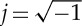. To be physically meaningful, the ‘frequency’ variable *f* takes on only positive, integer values (although to fully invert this transformation and recover the density in the original random variable, both positive and negative frequencies are needed). In the ECF domain indexed by frequency *f*, the representation of the density is much more compact than the density in the domain of the state variable *x*. This economy originates in the fact that the Fourier representation for highly periodic or close to periodic functions is sparse, that is, few of the Fourier coefficients are large in magnitude [42]. By contrast, the density in the domain of the original, untransformed variable will typically have no small values at all.

Given the typical sparsity of the ECF domain, we can simplify this by only retaining those coefficients that are larger in magnitude than a given threshold *λ*; the rest can be set to zero. Although simple, this procedure, called shrinkage or nonlinear thresholding in the statistical literature, is surprisingly powerful, in that (with very high probability) it is guaranteed to filter out the noise-only coefficients when the representation is sparse [42]. The choice of threshold *λ* can be made according to (minimax) statistical optimality principles, for example,
4.10
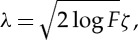
where *F* is the largest frequency of the ECF coefficients, *ζ* is the standard deviation of |*P*( *f*)| if these magnitudes are approximately Gaussian and *ζ*=1.482MAD(|*P*( *f*)|), where MAD is the median of the absolute deviations from the median of |*P*( *f*)|, if there are large outlier coefficients [15]. From these shrunken coefficients, the density of the states of the experimental system can be reconstructed using Fourier transform inversion
4.11
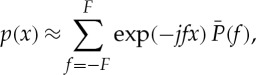
where 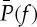 are the thresholded coefficients. The ECF shrinkage described above is a particularly efficient method for estimating multi-modal distributions with a large number of modes, where we know nothing more about the system other than it is periodic.

## 5. Example applications

### (a) Determining the dynamic periodicity of the bacterial flagellar motor

Many bacteria are motile and their method of movement involves the bacterial flagellum, a semi-rigid structure that protrudes from the cell wall. Flagella used for motion have the property, that when rotated in one direction, they function as an Archimedes screw. The flagellum is rotated by the BFM, a structure of about 45 nm, which is attached to the cell wall. At the small physical dimensions of the bacteria, the cellular liquid environment acts as if it is highly viscous. Therefore, the flagellum works like a screw propeller driving the bacteria forward. The motor has two essential parts: a stator and a rotor, which are constructed of multiple proteins arranged in a circular, periodic structure as described above. The energy to turn the rotor comes from an electrochemical gradient across the cellular membrane [43].

This extraordinary nanomachine is of considerable interest to biological physicists, who have devised special experimental assays to study the process of rotation of this motor. They are interested in asking questions such as whether the motor rotates in discrete steps or continuously, and if discrete, how many steps, and the functional process by which the motor changes direction [3,22].

**Figure 2.**
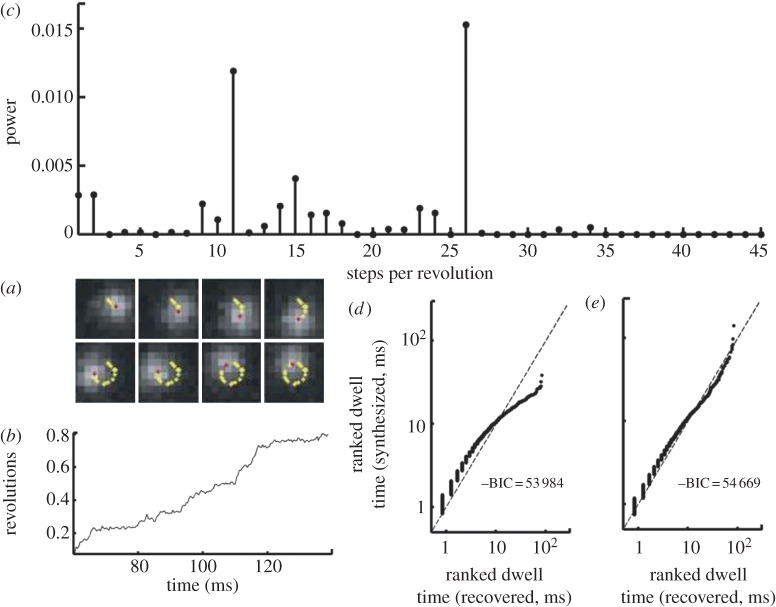
Extracting discrete states and dwell time distributions of the bacterial flagellar motor. (*a*) Sequence of EMCCD frames of a bead attached to a bacterial flagellar hook, the estimated centre of the bead over time shown in the yellow, time–position trace. (*b*) Time–angle signal estimated from the time–position trace in (*a*). (*c*) Square magnitude of ECF components averaged over step-smoothed time–position traces, showing the dominant 26-fold periodicity and strong evidence for 11-fold periodicity. This periodicity is related to the number of motor proteins. Horizontal axis is frequency in steps per revolution (*d*) Estimated dwell times against samples from an exponential model which assumes Poisson motor stepping, and (*e*) the same estimated dwell times against samples from a power-law model—(*d*) and (*e*) show quantile–quantile plots: a perfect model will have all points lying on the dotted diagonal line, and the closer the points to that line, the better the model for the dwell times. The power-law model is visually a much better fit, but additionally the Bayesian information criterion (BIC) that quantifies the quality of the fit is larger for the power-law model, confirming the visual conclusion that the motor stepping is non-Poisson. (Online version in colour.)

To address the question of the number of motor steps, a laser-illuminated 200 nm diameter bead was attached to the flagellar hook at the top of the BFM rotor [3]. The bead was imaged using an EMCCD camera at 2.4 kHz frame rate from which the angle of rotation of the motor was estimated by fitting a two-dimensional Gaussian function to the images (figure 2*a*). This resulted in a set of time–angle signals (example in figure 2*b*). The effects of the loading of the bead on the motor did not lead to statistically significant autocorrelation in the noise (or, which is equivalent, the sampling rate was too low to detect any autocorrelation). First, the signals were step-smoothed using total variation denoising equation (4.2) (see §4). After that, the distribution was estimated using the ECF method (equation (4.9); figure 2*c*). This led to a noisy distribution estimate, which was subsequently denoised using shrinkage with the threshold set in equation (4.10) with the MAD estimator. This signal processing clearly demonstrated that the BFM goes through 26 discrete conformational states during rotation, with superimposed 11-fold periodicity [15]. By applying shrinkage and inverting the ECF, the distribution of states can be found. Finally, an analysis of the dwell times of the conformational states during rotation showed that the previously held view of BFM stepping as a simple Poisson process (leading to exponentially distributed dwell times) is not supported by the data (figure 2*d*,*e*) [15].

### (b) Characterizing the catalytic steps in F1-ATPase

ATP is one of the most important molecules in cellular processes, being a nearly universal molecule for transporting energy between the different metabolic reactions of the cell. F1-ATPase complex is the rotary motor that forms the catalytic core of ATPase, the molecular system that uses a proton gradient to synthesize ATP. Alternatively, ATPase can operate backwards to hydrolyse ATP, generating a proton gradient. Using a similar illuminated bead assay as with the BFM above, biophysicists have been able to measure the rotation of the motor directly [15]. Similar questions about the rotation of this motor arise, including the number of discrete states, the existence or not of substepping between states, and the periodic arrangement and dwell times of those states.

**Figure 3.**
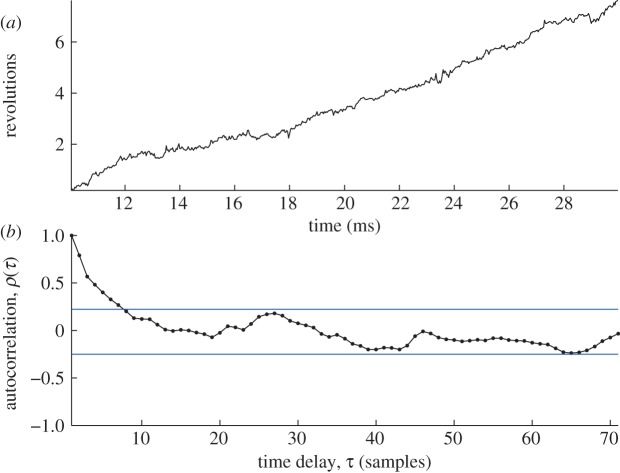
Quantifying F1-ATPase rotation. (*a*) Time–angle signal obtained by fitting a Gaussian to EMCCD frames of an illuminated bead attached to the molecule. (*b*) The autocorrelation of the time–angle signal showing clear evidence for Langevin dynamics since the autocorrelation at several of the low-order time delays is statistically significant (blue horizontal lines are the 95% confidence values assuming the null hypothesis of no autocorrelation). Extracting equilibrium states for this autocorrelated time series requires specialized signal processing techniques (see §4). (Online version in colour.)

As in the above experimental assay, EMCCD digital images at 30 kHz frame rate of a rotating 60 nm gold bead were analysed to extract an angle–time signal showing the rotation of the motor (figure 3*a*). In this case, statistically significant autocorrelation in the observation noise was detected indicating Langevin dynamics as in equation (2.1) (figure 3*b*). Therefore, equation (2.1) was discretized using a (first-order) numerical integration method, to arrive at a model for the dynamics [15]:
5.1
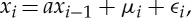
where *a* represents the feedback of past samples on the current sample of the signal, which introduces the autocorrelation. The forcing term *μ*_*i*_ consists of piecewise constant regions with instantaneous jumps at the state transitions of the molecular system. The term *ϵ*_*i*_ represents Gaussian noise due to thermal and illumination effects. This model can then be incorporated into equation (4.2) to create the following functional:
5.2
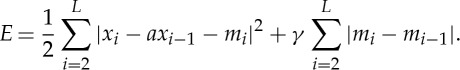
The feedback term *a* is estimated from the first autocorrelation coefficient of the noise in the experimental data. Minimizing this functional *E* with respect to the unknown piecewise constant signal *m* is then carried out using quadratic programming [30]. Having obtained the step-smoothed conformational states of the motor by minimizing equation (5.2) as above, the distribution of states was estimated using the ECF method (equation (4.9)). This showed the dominant periodicity of the motor to be sixfold validating known models for this enzyme. Subsequent examination of the distribution of dwell times of the conformational states revealed by this signal processing analysis showed strong evidence for the existence of a pair of cascading rate-limiting substeps [15].

## 6. Summary and conclusions

This paper has outlined the topic of biophysical DSP, first by describing the scientific context in which biophysical signals are generated, and explaining the specific nature of a wide class of biophysical signals. Then the limitations of classical linear time invariant DSP in this application were discussed, concluding that there is an inherent need for nonlinear and non-Gaussian DSP algorithms. This motivated the introduction of piecewise constant filtering, and techniques for handling multi-modal and periodic distributions. Finally, example applications were described in detail.

The problems of biophysical DSP are particularly challenging because of the need to process non-smooth multi-modal time series with autocorrelated noise, and the discipline is immature. Thus, there is much room for exploration and discovery. As an example of unexplored territory, one might want to go beyond point estimates, that is, only a single result is produced, rather than a distribution of results (perhaps summarized into a confidence interval). This is a limitation because a single answer to a scientific question, even if it is the most likely one, does not convey the full uncertainty due to the sources of error that are inevitable in all experimental situations. For example, it is useful in many circumstances to apply Bayesian reasoning so that prior information can be formalized and incorporated into the computation of the posterior distribution of the output.

The aim of this paper has been to introduce the mathematics and applications of this emerging research topic in an inclusive way; however, any single paper surveying this topic is bound to miss out on important research. For example, this paper has only touched upon parts of the extensive field of biophysical digital image processing, which is of critical importance in a wide array of experimental applications. Nonetheless, we can confidently claim that the field of biophysical DSP is set to become more important over time as science seeks to uncover more and more of the fundamental mechanisms of life at the cellular and molecular scale. To encourage further experimentation, software implementations of the signal processing algorithms described in this paper are available upon request from M.A.L or from our respective websites.
